# Effect of light‐load resistance exercise on postprandial amino acid transporter expression in elderly men

**DOI:** 10.14814/phy2.13444

**Published:** 2017-09-28

**Authors:** Jakob Agergaard, Jacob Bülow, Jacob K. Jensen, Søren Reitelseder, Andreas Bornø, Micah J. Drummond, Peter Schjerling, Lars Holm

**Affiliations:** ^1^ Institute of Sports Medicine Copenhagen Department of Orthopedic Surgery M Bispebjerg Hospital Copenhagen Denmark; ^2^ Center for Healthy Ageing Faculty of Health Sciences University of Copenhagen Copenhagen Denmark; ^3^ Department of Physical Therapy University of Utah Salt Lake City Utah; ^4^ Clinical Metabolomics Core Facility Rigshospitalet, Copenhagen Denmark; ^5^ Department of Biomedical Sciences Faculty of Health and Medical Sciences University of Copenhagen Copenhagen Denmark

**Keywords:** Amino acid transporter, light load, resistance exercise, sarcopenia, whey protein

## Abstract

An impaired amino acid sensing is associated with age‐related loss of skeletal muscle mass. We tested whether light‐load resistance exercise (LL‐RE) affects postprandial amino acid transporter (AAT) expression in aging skeletal muscle. Untrained, healthy men (age: +65 years) were subjected to 13 h of supine rest. After 2 1/2 h of rest, unilateral LL‐RE was conducted (leg extensions, 10 sets of 36 repetitions) at 16% 1RM. Thereafter, the subjects were randomized into groups that orally ingested 40 g of whey protein either as hourly drinks (4 g per drink) (PULSE,* N* = 10) or two boluses (28 g at 0 h and 12 g at 7 h) (BOLUS,* N* = 10), or hourly isocaloric maltodextrin drinks (placebo, *N* = 10). Quadriceps muscle biopsies were taken at 0, 3, 7, and 10 h postexercise from both the resting and exercised leg, from which the membrane protein and mRNA expression of select AATs were analyzed by Western Blot and RT‐PCR, respectively. LAT1 and PAT1 protein expression increased in response to LL‐RE in the PULSE group, and SNAT2 and PAT1 protein expression increased in the BOLUS group when plasma BCAA concentration was low. In all three groups, LL‐RE increased LAT1 mRNA expression, whereas a time course decrease in SNAT2 mRNA expression was observed. LL‐RE increased membrane‐associated AAT protein expression and mRNA expression. Altered AAT protein expression was only seen in groups that ingested whey protein, with the greatest effect observed after hourly feeding. This points toward an importance of AATs in the anabolic response following LL‐RE and protein intake.

## Introduction

Feeding protein or free amino acids to adults is known to stimulate muscle protein synthesis (MPS) (Bohé et al. 2001). The link between peripheral hyperaminoacidemia and increased MPS results from cells sensing the increased amino acid concentration, increased amino acid uptake, and intracellular amino acid utilization for protein translation (Bohé et al. 2003). The molecular link between hyperaminoacidemia and increased MPS is the activation of the mechanistic target of rapamycin complex 1 (mTORC1) signaling pathway (Fujita et al. 2007; Dreyer et al. 2008; West et al. 2011; D'Souza et al. 2014). Activation of mTORC1 occurs when the complex anchors to the lysosomal membrane (Bar‐Peled and Sabatini 2014). Among numerous upstream signals responsible for mTORC1 activation, three amino acid transporters (AATs) are believed to play a central role: L‐type amino acid transporter 1 (LAT1)/solute‐linked carrier (SLC) 7A5, sodium‐coupled neutral amino acid transporter 2 (SNAT2)/SLC38A2, and proton‐assisted amino acid transporter 1 (PAT1)/SLC36A1 (Poncet and Taylor 2013). In addition to being an important transporter of branched‐chain amino acids (BCAAs), especially leucine, LAT1 has a catalytic subunit that is involved in nutrient signaling and mTORC1 activation (Poncet et al. 2014). Furthermore, intracellular leucine is proposed to stimulate leucyl tRNA synthetase (LRS) binding to the Rag‐GTPase that is involved in mTORC1 activation (Han et al. 2012). SNAT2 transports glutamine into the cell, which is needed for the exchange transport of BCAAs by LAT1. Additionally, downstream SNAT2 signaling is believed to be directly involved in mTORC1 activation (Pinilla et al. 2011), although the exact mechanism is unknown. PAT1 is important at the lysosomal membrane, where it is proposed to be involved in vacuolar H(+)‐ATPase (v‐ATPase) activity toward Ragulator, which together with the Rag complex, is a central docking molecule for mTORC1 at the surface of the lysosome (Zoncu et al. 2011; Ögmundsdóttir et al. 2012). It is unknown whether AATs are rate limiting for mTORC1 activation, but intracellular amino acid availability (Beugnet et al. 2003) and especially the leucine concentration and repletion capacity (Schriever et al. 2013) are related to mTORC1 signaling.

Aging increases skeletal muscle anabolic resistance, as shown by a decreased (Cuthbertson et al. 2005) and delayed (Drummond et al. 2008) response to anabolic stimuli, such as resistance exercise and amino acid availability. The cellular change in advancing age that leads to anabolic resistance is not yet fully understood. However, decreased expression of AATs has been proposed (Drummond et al. 2011), which would affect amino acid sensing and subsequent signaling (Guillet et al. 2004; Cuthbertson et al. 2005). Potent interventions to increase the expression of AATs could, therefore, imply a greater sensitivity toward amino acids and, thus, attenuate anabolic resistance. It has been shown that resistance exercise coupled with amino acid ingestion increases AAT protein expression in young subjects (Drummond et al. 2011; Dickinson et al. 2013) and AAT mRNA expression in young (Churchward‐Venne et al. 2012; Reidy et al. 2014) and older subjects (Dickinson et al. 2014). Moreover, it has been shown that increased amino acid availability alone increases AAT protein (Drummond et al. 2010) and mRNA expression (Drummond et al. 2010; Churchward‐Venne et al. 2012). Studies comparing the response to resistance exercise and essential amino acid (EAA) intake in young versus older subjects show different patterns in AAT expression, which suggests that aging affects the AAT response (Drummond et al. 2011; Dickinson et al. 2013).

Although most studies of skeletal muscle showed a robust change in AAT mRNA expression in response to anabolic stimuli (Churchward‐Venne et al. 2012; Dickinson et al. 2014; Reidy et al. 2014), some studies showed no, or very little, change in AAT protein expression (Drummond et al. 2011; Reidy et al. 2014). Thus far, studies of AAT protein expression in human skeletal muscle have performed Western blotting on whole‐muscle homogenates. However, AATs incorporated into the membrane through which they transport amino acids are of the utmost importance. Posttranslational modification and translocation of AATs have been suggested to occur (Roos et al. 2009). Thus, fractionating muscle homogenates and extracting the membrane fraction prior to Western blotting would elucidate changes in AAT membrane expression, thereby providing a clearer picture of changes in the potential of amino acid sensing of the muscle cells.

Resistance exercise with a load equivalent to 70–80% of the one‐repetition maximum (1RM) is normally recommended to induce muscle hypertrophy. However, recent studies have shown hypertrophic and anabolic responses even from resistance exercise at a light load (LL‐RE) in young (Holm et al. 2008; Burd et al. 2010; Bechshoeft et al. 2013) and elderly subjects (Devries et al. 2015; Murphy et al. 2015; Agergaard et al. 2017). Challenging the hypertrophic response by resistance exercise at a low percentage of the 1RM is intriguing, as this could be a well tolerable and applicable exercise for elderly and frail individuals to counteract age‐related muscle atrophy. The question is whether the anabolic response from LL‐RE could be ascribed to a greater skeletal muscle sensing of nutrients.

Therefore, the purpose of the present study was to investigate how resistance exercise with light loads and protein intake provided via a frequent or bolus feeding regimen alters the expression of membrane‐associated AATs. Moreover, AAT mRNA expression was measured to elucidate the potential for de novo AAT synthesis. We hypothesized that LL‐RE would increase the protein and mRNA expression of AATs, and that a greater effect would be seen in groups receiving protein, albeit in a pattern reflecting the feeding regime.

## Materials and Methods

### Ethical approval

Participants were informed about the study protocol, the risks of tests and investigations, and their rights according to the Declaration of Helsinki II before volunteering for the project. The study protocol was approved by the Research Ethics Committee Region Hovedstaden Committee D (H‐2‐2012‐041, 13 June 2012). All participants gave written informed consent.

### Subjects

Healthy elderly male subjects >65 years of age were included through web and newspaper advertisement. Subjects were excluded if they were smokers, had relatives with type II diabetes, received daily medications or supplements that could have an impact on muscle protein synthesis, had an alcohol intake >21 units per week, had a body mass index (BMI) >28, or regularly performed exercise or did strenuous physical work. Randomization of 30 subjects was double‐blinded into one of three groups; a control group that ingested an isocaloric carbohydrate hourly (placebo) (*N* = 10), a protein group that ingested protein hourly (the PULSE group) (*N* = 10), or a protein group that ingested protein in two doses (the BOLUS group) (*N* = 10). The groups were homogenous in regard to age, height, weight, BMI, fat‐free mass (FFM), and 1RM muscle strength (Table 1). Data from these subjects have recently been published (Agergaard et al. 2017).

**Table 1:** Subject characteristics at baseline for the placebo, PULSE, and BOLUS groups. Data are shown as the mean ± SD. Subject characteristics have previously been published (Agergaard et al. 2017)

	Placebo *N* = 10	PULSE *N* = 10	BOLUS *N* = 10	*P*‐value
Age (years)	73.0 ± 4.7	71.6 ± 4.3	69.7 ± 4.7	0.28
Height (m)	1.78 ± 0.06	1.75 ± 0.07	1.77 ± 0.06	0.55
Weight (kg)	80.7 ± 6.8	79.8 ± 11.8	79.5 ± 9.5	0.96
BMI (kg m^−2^)	25.4 ± 1.5	26.0 ± 3.0	25.2 ± 1.9	0.71
FFM (kg)	59.1 ± 5.9	57.6 ± 4.7	57.8 ± 4.7	0.76
1RM of Ex leg (kg)	44.6 ± 10.8	43.0 ± 6.1	42.7 ± 6.7	0.86

### Inclusion

Body composition was measured by a dual X‐ray absorptiometry (DXA) scan, and blood samples were taken for a general health screening at inclusion. Moreover, unilateral quadriceps muscle strength was measured in a leg extension device (Technogym, Super Executive Line, Gambettola, Italy) by a 3RM test from which the 1RM was estimated using the Brzycki equation (Brzycki 1995). The leg extensions were performed in a 90° range‐of‐motion with a knee‐joint angle from 100° to 10°. After a brief warm‐up on a Monarch cycle for 5 min, the subjects performed three sets of five leg extensions at a light load before the test in the leg extension device. During the test, the subjects were instructed to perform four repetitions. To avoid fatigue, the subjects were stopped after two repetitions if they could easily perform the leg extensions, and more weight was added. When they were able to perform only three, but not four, repetitions, the load was noted as the 3RM.

### Trial day

Prior to the trial day, the subjects were instructed not to perform any strenuous physical exercise or work, ingest sufficient protein, and refrain from alcohol‐ and caffeine‐containing substances for 1 week or 1 day, respectively, before the trial day as previously described (Agergaard et al. 2017). After overnight fasting, the subjects arrived at the Institute of Sports Medicine Copenhagen, Bispebjerg Hospital in the morning by taxi to avoid any physical activity.

**Figure 1:**
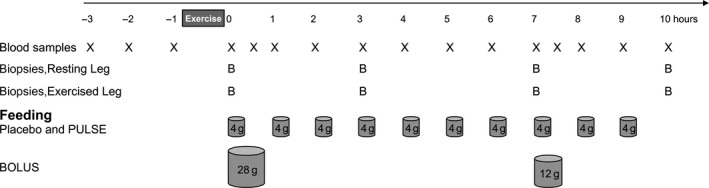
Study design of the 13‐h trial period. X denotes blood sampling, B denotes muscle biopsy sampling and cylinders denote time and the amount of placebo or protein intake in the three groups.

After arrival, the subjects rested in the supine position, and venous catheters were inserted retrograde into the antecubital veins of both arms. At time point −45 min, the LL‐RE commenced (Fig. 1). The subjects performed the exercise using the same device and at the same setting as for the 3RM test. The exercise consisted of unilateral leg extensions with 10 sets of 36 repetitions at 16% of their 1RM. Subjects were randomized to exercise with either their strongest or weakest leg. Each repetition lasted 5 sec: 2 sec in the concentric phase and 3 sec in the eccentric phase, including a brief detensioning of the exercising muscle before the next repetition. A break of 30 sec was given between each set. In total, the exercise lasted for 35 min. The contralateral leg remained at rest.

The subjects returned to rest in the supine position after the last exercise set. Thereafter, they were given the first drink at time point 0 h (Fig. 1). In total, the PULSE and BOLUS groups consumed equal amounts of protein being 700 mg × kg FFM^−1^ of whey protein isolate (40.4 g on average) (LACPRODAN DI‐9224, Arla Foods Ingredients Group P/S, Denmark). The PULSE group consumed whey protein drinks hourly (70 mg whey protein × kg FFM^−1^, on average 4 g per drink) to keep an elevated availability of AAs throughout the 10‐h postexercise period. The BOLUS group ingested the whey protein in two boluses; the first drink containing 490 mg of whey protein × kg FFM^−1^, on average 28.3 g of whey protein, at 0 h, and the second drink containing 210 mg of whey protein × kg FFM^−1^, on average 12.1 g of whey protein, at 7 h. The PULSE group design is to a great degree academic. However, importantly the PULSE group was included in the present study to make comparisons on myofibrillar protein synthesis (MPS) presented in Agergaard et al. (2017) with the study on young men by Bechshoeft et al. (2013). In addition, the BOLUS group was designed with a more realistic approach toward protein feeding. The protein provision in the BOLUS group was skewed (28 g at 0 h and 12 g at 7 h) in order to match the amount of protein ingested in the PULSE group within the same time period (28 g in total from 0–7 h and 12 g in total from 7–10 h). The placebo group consumed 665 mg × kg FFM^−1^ (isocaloric) maltodextrin, approximately 38 g in total (Glucidex IT19, Roquette, Clement Lise, France), which was given in the same hourly feeding pattern as that of the PULSE group (Fig. 1). Beside these drinks, the subjects were only allowed to consume water throughout the study period.

### Muscle biopsies

At 0, 3, 7, and 10 h, muscle biopsies were taken from each leg. The muscle biopsies were obtained from the lateral part of the vastus lateralis muscle with a 4‐mm biopsy needle (Bergström, Stockholm, Sweden). Briefly, the skin was shaved and disinfected before local anesthetization (1% lidocaine). Immediately before biopsy sampling, the placebo or protein drink was administered. An incision was made through which a muscle biopsy was taken. Thereafter, the incision was strapped with SteaStrips and covered with waterproof plaster. Subsequently, the thigh muscle was compressed for 30 min with an elastic band and a compression pad at the incision site to avoid an intramuscular hematoma. A new incision was made for each biopsy, with approximately 3 cm between each incision. The incisions were made in a randomized order between subjects, but in the same order for the exercised and resting leg within subjects. When taken, the muscle specimen was quickly freed from any visible blood, fat, and connective tissue, frozen in liquid N_2_ and stored at −80°C until further analysis.

### Amino acid concentrations

Plasma amino acid (AA) concentrations were measured from blood samples taken at baseline immediately after exercise (0 h) and at 0.5, 1, 2, 3, 5, 7, 7.5, 8, 9, and 10 h into the postprandial period. Liquid chromatography tandem mass‐spectrometry (LC‐MS/MS) (TSQ Vantage; Thermo Fisher Scientific, San Jose, CA) was used to measure the amino acid concentrations as described elsewhere (Bornø and van Hall 2014). For the analysis, 150 *μ*L of plasma was used. Importantly, each of the individual 19 amino acids was quantified using its own stable isotopically labeled internal standard (uniformly labeled‐^13^C/^15^N). Because of the substantial cost of the LC‐MS/MS analysis, the number of time points was reduced in the three groups, but in a fashion that matched the time points where changes would be expected. Thus, in the placebo group, amino acid concentrations were measured at 0, 0.5, 1, 2, 6, and 10 h, while those of the PULSE group was measured at 0, 0.5, 1, 2, 4, 6, 8, and 10 h, and those of the BOLUS group were measured at 0, 0.5, 1, 2, 3, 5, 7, 7.5, 8, 9, and 10 h. A flood‐primed (1320 mg unlabeled phenylalanine and 165 mg of ring‐^13^C_6_‐phenylalanine, Cambridge Isotopes Laboratories, Andover, MA) continuous infusion (ring‐^13^C_6_‐phenylalanine 8.0 *μ*mol × kg FFM^−1^ × h^−1^) of a phenylalanine tracer was applied to measure the fractional synthetic rate, which is presented elsewhere (Agergaard et al. 2017). Thus, phenylalanine was withdrawn from the calculations of EAAs and total AAs so that the AA data would solely reflect the different feeding regimens.

### Muscle tissue fractionation

Skeletal muscle tissue samples were fractionated into membrane and cytosolic fractions. Approximately 30 mg of frozen skeletal muscle tissue per sample was homogenized. The muscle specimen was placed into a cooled glass tube (Pyrex glass tube, Corning, NY) and homogenization buffer was added (10 *μ*L mg^−1^ tissue). The homogenization buffer consisted of 25 mmol L^−1^ Tris‐HCl, 0.5 mmol L^−1^ EGTA, 2 mmol L^−1^ EDTA, 1 mmol L^−1^ PMSF, 1 mmol L^−1^ DTT, and 300 mmol L^−1^ sucrose, pH 7.2. Immediately before using the homogenization buffer, a 1:100 dilution of a phosphatase inhibitor cocktail (Calbiochem, no. 524624, Merck Millipore, Billerica, MA), 20 *μ*mol L^−1^ leupeptin and 120 *μ*mol L^−1^ pepstatin A was added. The sample was homogenized with a glass pestle (Pyrex glass pestle tissue grinder, Corning) mounted on a tabletop homogenizer (Eurostar Power Basic, IKA Works Inc., Wilmington, NC), transferred to a separate tube and centrifuged at 1000***g*** for 15 min at 4°C. Subsequently, the supernatant containing cytosolic proteins was transferred to a cooled ultracentrifuge tube (Beckman Coulter Inc., no. 343778, Brea, CA) and used for other analyses. To the pellet containing the membrane fraction, homogenization buffer (160 *μ*L) containing 1% IGEPAL 680 was added. The pellet was resuspended by vortex and transferred to a cooled ultracentrifuge tube and incubated for 30 min. Subsequently, the sample was centrifuged at 100,000***g*** for 30 min at 4°C. After centrifugation, the supernatant was transferred to new Eppendorf tubes for further analysis, and the protein concentration was quantified with a Bradford‐based microplate protein assay (#500‐0006, Bio‐Rad, Hercules, CA); the protein yield was 4.75 ± 0.97 *μ*g mg^−1^ (mean ± SD) of wet‐weight muscle tissue. The purity of the fractions was validated by blotting for Caveolin‐1 (membrane protein), Insulin Receptor *β* (membrane protein), and GAPDH (cytosolic protein) on both membrane and cytosolic fraction (data not shown).

### Western blotting

Western blotting was performed on the membrane fraction to measure the expression of the AATs. Membrane fraction samples were diluted 1:1 in 2× sample buffer (1.25 mol L^−1^ Tris pH 6.8, 25% v/v glycerol, 2.5% SDS, 2.5% v/v *β*‐mercaptoethanol and 0.2% w/v bromophenol blue). Samples were heated at 95°C for 3 min prior to loading on SDS‐PAGE gels. Five micrograms of protein was loaded per well on 26‐well 15% Criterion Tris‐HCl precast gels (Bio‐Rad) and resolved by SDS‐PAGE for 1 h at 150 V in electrophoresis buffer (25 mmol L^−1^ Tris‐Base, 190 mmol L^−1^ glycine and 3.5 mmol L^−1^ SDS). Proteins were blotted by wet transfer (#170‐3912, Bio‐Rad) to PVDF membranes (Bio‐Rad) for 1 h at 50 V in transfer buffer (10 mmol L^−1^ CAPS pH 11.0 and 10% v/v methanol). Membranes were blocked for 45 min at room temperature with 2% skimmed milk powder in Tris‐buffered saline (TBS) containing Tween‐20 (TBST). The membranes were incubated with primary antibodies overnight at 4°C in a solution of 2% skimmed milk powder in TBST. The primary antibodies were LAT1 (ab85226; Abcam, Cambridge, MA) diluted 1:1000, SNAT2 (sc‐67081; Santa Cruz Biotechnology, Dallas, TX) diluted 1:2000, and PAT1 (ab110513; Abcam) diluted 1:500. The next morning, the membranes were washed in TBST and secondary anti‐rabbit antibody (sc‐2313, Santa Cruz Biotechnology) diluted 1:6000 in 2% skimmed milk powder in TBST was added to the membranes and incubated for 1 h at room temperature. Subsequently, the membranes were washed in TBST, incubated with chemiluminescence reagent (ECL Plus, GE Healthcare, Pittsburgh, PA) for 5 min, and visualized on a chemiluminescence imager (ChemiDoc XRS+, Bio‐Rad). Band intensities were quantified using ImageJ (National Institutes of Health, Bethesda, MD).

Ponceau‐S staining of the membranes was used to control that equal amounts of protein had been loaded onto the gels before SDS‐PAGE by evaluating the Ponceau‐S staining of a selected band of interest from the samples of each subject. Membranes were stained with a Ponceau‐S solution for 5 min at room temperature and washed twice for 5 min in dH_2_O. Images of the membranes were obtained (ChemiDoc XRS+, Bio‐Rad) and band intensities were quantified using ImageJ (National Institutes of Health). Instead a membrane‐specific housekeeping protein could have been used as loading control. However, since the current study includes both exercise and nutritional interventions, applying a membrane‐specific protein as housekeeping protein could fail to be reliable, as the expression must be stable regardless of the intervention but also regardless of time (not affected by diurnal fluctuation). Thus, Ponceau‐S staining was applied in the current study.

### RT‐PCR

Reverse transcription‐PCR was used to measure the mRNA expression of LAT1, SNAT2, and PAT1. RNA was purified with TriReagent (RT 118; Molecular Research Center, Cincinnati, OH) from approximately 10 mg of muscle tissue as previously described (Agergaard et al. 2015). cDNA synthesis was performed using Omniscript reverse transcriptase (Qiagen, Hilden, Germany), using 250 ng of muscle RNA and amplified with specific primers (0.1 *μ*mol L^−1^ each, Table 2) as previously described (Agergaard et al. 2015).

**Table 2:** Primers for RT‐PCR analyses of housekeeping genes and AA

Target	Sense primer	Antisense primer	Ref. sequence
RPLP0	GGAAACTCTGCATTCTCGCTTCCT	CCAGGACTCGTTTGTACCCGTTG	NM_053275.3
GAPDH	CCTCCTGCACCACCAACTGCTT	GAGGGGCCATCCACAGTCTTCT	NM_002046.4
LAT1	GTGGCTCCTCCAGGGCATCT	CTCGGCCTCCTGGCTATGTCTC	NM_003486.5
SNAT2	AAACACCACCTTAACACAGCCAACA	TGAAAAGATCAGAATTGGCACAGCA	NM_018976.4
PAT1	ACCCCAGCCACCTCCCCTTG	GAACTTCCGAGGATCCTTCATTTTG	NM_078483.2

**Figure 2:**
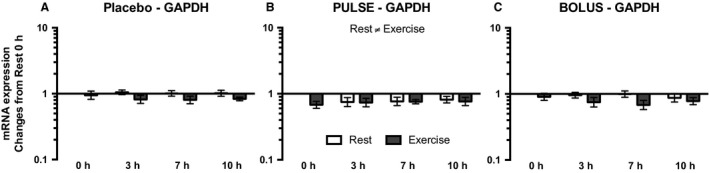
GAPDH mRNA expression for the placebo (A), PULSE (B), and BOLUS (C) groups for the resting leg (white bars) and exercised leg (dark gray bars) at 0, 3, 7, and 10 h postexercise. Data are normalized to RPLP0 mRNA expression and expressed as changes from 0 h in the resting leg. Data are shown as the GeoMean ± back‐transformed SEM.

The housekeeping genes glyceraldehyde 3‐phosphate dehydrogenase (GAPDH) and ribosomal protein large P0 (RPLP0) served as controls. When normalizing the level of GAPDH to that of RPLP0, an overall decrease in GAPDH expression was observed in the exercised leg compared with the resting leg in the PULSE group (*P* = 0.048) (Fig. 2B), and a tendency toward a difference in the expression of GAPDH and RPLP0 between legs was seen in the ANOVA testing in the placebo (*P* = 0.063) and BOLUS groups (*P* = 0.084) (Fig. 2A and C). We do not believe that the scale of the quick decrease in GAPDH expression normalized to RPLP0 that seemed to appear at 0 h in response to exercise in the PULSE group was due to an immediate synthesis of RPLP0, but rather due to degradation of GAPDH mRNA. Therefore, we expect changes in GAPDH mRNA expression, normalized to that of RPLP0, to be caused by changes in GAPDH mRNA expression; thus, RPLP0 was chosen as the housekeeping gene, and all mRNA expression data were normalized to RPLP0 expression.

### Statistics

The normality of data was assessed by Shapiro–Wilk normality test. However, due to a low sample size (*N*), these normality tests would sometimes fail. In that case, histogram was plotted to visually assess normality and skewness.

Subject characteristics were analyzed by one‐way ANOVA using Prism 6 (GraphPad Software, La Jolla, CA).

Plasma AA concentrations were log‐transformed before being subjected to ANOVA analysis, as they would otherwise not be normally distributed. One‐way ANOVA testing was performed within groups with a post hoc test (Dunnett's test) to reveal changes from the baseline. Because the measured plasma AA concentrations for the placebo and PULSE groups seemed to be linearly expressed after 1 h, a linear regression analysis was performed using Prism 6 (GraphPad Software) on the levels measured from 1 to 10 h to reveal whether the plasma AA levels changed over time.

Two‐way ANOVA with repeated measures for time were conducted on plasma leucine and BCAA concentrations for time points common to all three groups. Whenever two‐way ANOVA testing showed significant differences (*P* < 0.05), post hoc tests (the Bonferroni procedure) were performed.

The area under the curve (AUC) for plasma leucine and BCAA were calculated from the available time points. If blood could have been drawn every half hour, multiple peaks in the plasma leucine and BCAA concentrations would have been revealed in the PULSE group after each drink. Thus, the total AUC for the PULSE group was underestimated. Plasma leucine and BCAA AUCs were analyzed by one‐way ANOVA with a post hoc test (Student–Newman–Keuls test) whenever ANOVA testing showed significant differences (*P* < 0.05).

Amino acid transporter protein and mRNA expression were subjected to two‐way ANOVA with repeated measures for leg and time within group to analyze the effect of LL‐RE. Whenever ANOVA testing showed significant differences (*P* < 0.05), a post hoc test (Student–Newman–Keuls test) was performed.

Unless otherwise stated, Sigma Plot version 12.0 (Systat Software Inc., San Jose, CA) was used for the statistical analysis. All plasma AA data are shown as the mean ± SEM. Before ANOVA testing, protein and mRNA expression were log‐transformed, due to the logarithmic nature of the data after normalized to the resting leg at 0 h. Protein and mRNA data are shown as the geometric mean (GeoMean) ± back‐transformed SEM. A *P* < 0.05 was considered to be statistically significant.

## Results

### Amino acid concentrations

All measurements of plasma concentrations for each individual BCAA (leucine, valine, and isoleucine) during the 10‐h postprandial period were higher than those at the baseline (0 h) (*P* < 0.05) for the PULSE and BOLUS groups (Table 3). Linear regression on measurements made from 1 h onwards showed that the plasma concentrations in the PULSE group increased over time for leucine (*R* ^2 ^= 0.6039, *P* < 0.0001), valine (*R* ^2 ^= 0.4382, *P* < 0.0001), and isoleucine (*R* ^2 ^= 0.6112, *P* < 0.0001) (Table 3).

**Table 3:** Plasma AA concentrations (*μ*mol L^−1^) during the 10‐h postprandial period for the placebo, PULSE, and BOLUS groups. *R* ^2^ and *P*‐values show the results of the linear regression test in the placebo and PULSE groups. Results of Dunnett's post hoc test are shown by *, which denote a significant difference from 0 h (*P* < 0.05) within the placebo, PULSE, or BOLUS groups

		0 h	0.5 h	1 h	2 h	3 h	4 h	5 h	6 h	7 h	7.5 h	8 h	9 h	10 h	*R* ^2^	*P*‐value
Aspartic acid	Placebo	2.12 ± 0.27	2.21 ± 0.24	2.48 ± 0.27	2.15 ± 0.36				1.94 ± 0.29					2.42 ± 0.53	0.0005	0.8944
	PULSE	3.91 ± 1.88	2.91 ± 0.45	2.00 ± 0.22	1.83 ± 0.27		2.13 ± 0.57		2.31 ± 0.45			1.78 ± 0.26		2.11 ± 0.45	0.0005	0.8736
	BOLUS	2.75 ± 0.47	12.44 ± 2.34*	14.49 ± 2.47*	4.86 ± 0.60	2.16 ± 0.35		1.73 ± 0.21		1.92 ± 0.27	8.96 ± 2.49*	7.07 ± 0.86*	2.18 ± 0.29	2.93 ± 1.08		
Glutamic acid	Placebo	37.4 ± 5.0	42.4 ± 6.3	41.7 ± 5.9	35.7 ± 4.8				34.4 ± 4.6					30.4 ± 4.4	0.0597	0.1511
	PULSE	33.8 ± 3.1	38.7 ± 4.8	32.7 ± 3.3	31.9 ± 3.4		30.8 ± 3.5		32.9 ± 3.6			28.7 ± 3.1		26.5 ± 2.3*	0.0397	0.1339
	BOLUS	42.3 ± 5.8	68.6 ± 10.9*	73.8 ± 11.3*	51.9 ± 6.5	39 ± 6.9		34.6 ± 3.7		33.3 ± 5.2*	57.8 ± 12.6	59.1 ± 7.0*	42 ± 6.7	36.8 ± 4.8		
Serine	Placebo	90.3 ± 5.0	89.9 ± 5.0	91.4 ± 4.5	88.3 ± 3.9*				89.3 ± 5.0					88.5 ± 5.6	0.0023	0.7811
	PULSE	83.8 ± 3.1	99.6 ± 4.6*	92.8 ± 4.2*	94.2 ± 4.2*		93.1 ± 4.7*		94.3 ± 3.8*			90.7 ± 3.3*		95.5 ± 3.6*	0.0002	0.9265
	BOLUS	88.0 ± 2.7	140.8 ± 9.9*	142.7 ± 8.0*	104.0 ± 4.5*	86.4 ± 3.7		82.8 ± 3		85.2 ± 3.0	129.6 ± 6.5*	112.4 ± 4.2*	86.3 ± 2.8	93.4 ± 6.0		
Glycine	Placebo	182.2 ± 12.4	177.4 ± 12.6	179.5 ± 14.5	177.9 ± 12.9				180.1 ± 11.1					184.3 ± 12.2	0.0030	0.7526
	PULSE	185.0 ± 7.9	187.0 ± 9.7	183.4 ± 8.9	178.0 ± 7.4		175.3 ± 7.8*		167.8 ± 6.4*			156.1 ± 6.1*		159.6 ± 6.6*	0.1614	0.0018
	BOLUS	193.7 ± 11.1	220.0 ± 12.9*	209.2 ± 13.2	165.1 ± 7.5*	156.4 ± 8.8*		163.6 ± 7.8*		172.0 ± 7.6*	186.9 ± 10.2	167.4 ± 9.0*	150.0 ± 7.6*	171.8 ± 10.2*		
Asparagine	Placebo	33.8 ± 1.4	32.9 ± 1.4	33.0 ± 1.5	32.4 ± 1.3				33.0 ± 1.5					32.5 ± 1.4	0.0003	0.9245
	PULSE	31.2 ± 1.1	39.1 ± 2.5*	36.4 ± 1.9*	37.8 ± 2.1*		36.5 ± 2.3*		38.3 ± 2.4*			37.2 ± 2.3*		39.7 ± 2.4*	0.0143	0.3712
	BOLUS	30.6 ± 1.0	59.0 ± 5.5*	62.6 ± 5.0*	42.5 ± 2.4*	33.1 ± 1.7		30.2 ± 1.6		30.5 ± 1.4	53.2 ± 4.2*	46.5 ± 2.5*	31.5 ± 1.2	32.4 ± 1.5		
Glutamine	Placebo	551 ± 23	546 ± 22	560 ± 23	556 ± 24				567 ± 20					542 ± 26	0.0048	0.6892
	PULSE	514 ± 24	561 ± 27*	578 ± 26*	576 ± 23*		576 ± 26*		586 ± 26*			568 ± 23*		575 ± 21*	0.0004	0.8746
	BOLUS	530 ± 25	625 ± 32*	652 ± 37*	559 ± 25	517 ± 36		542 ± 31		541 ± 31	613 ± 39*	601 ± 30*	495 ± 22	532 ± 22		
Histidine	Placebo	76.2 ± 2.3	80.5 ± 3.9	75.6 ± 2.2	75.4 ± 2.1				77.1 ± 2.3					72.2 ± 3.2	0.0193	0.4196
	PULSE	72.1 ± 2.1	77.7 ± 2.1	76.6 ± 2.5	80.6 ± 3.1*		80.1 ± 2.8*		83.5 ± 3.7*			79.0 ± 2.2*		83.5 ± 2.8*	0.0304	0.1909
	BOLUS	68.0 ± 2.9	86.0 ± 3.6*	91.3 ± 4.3*	75.1 ± 2.4*	67.7 ± 2.4		68.3 ± 2.3		70.1 ± 2.1	83.1 ± 3.7*	83.8 ± 3.4*	64.5 ± 2.4	68.1 ± 3.0		
Threonine	Placebo	117.5 ± 4.9	113.7 ± 4.3	111.6 ± 5.3	106.8 ± 5.5*				103.2 ± 4.6*					99.3 ± 4.7*	0.0792	0.0963
	PULSE	106.2 ± 4.7	134.6 ± 8.6*	127.1 ± 7.5*	138.8 ± 7.4*		142.4 ± 7.7*		152.9 ± 7.0*			153.2 ± 7.1*		165.0 ± 9.0*	0.2134	0.0003
	BOLUS	107.5 ± 5.8	210.9 ± 20.8*	238.7 ± 20.0*	189.0 ± 12.3*	153.8 ± 8.1*		131.4 ± 7.9*		122.3 ± 6.2	205.9 ± 16.0*	188.3 ± 10.7*	138.5 ± 6.4*	133.5 ± 6.3*		

		0 h	0.5 h	1 h	2 h	3 h	4 h	5 h	6 h	7 h	7.5 h	8 h	9 h	10 h	*R* ^2^	*P*‐value
Alanine	Placebo	345.0 ± 21.7	322.6 ± 22.6	305.8 ± 21.8	297.7 ± 19.1*				288.0 ± 18.2*					267.2 ± 23.0*	0.0538	0.1734
	PULSE	278.1 ± 14.2	298.1 ± 12.7	292.3 ± 14.7	291.1 ± 11.7		284.2 ± 10.9		282.4 ± 12.1			273.2 ± 11.6		284.0 ± 12.7	0.0167	0.3342
	BOLUS	261.7 ± 16.7	353.5 ± 33.7*	399.8 ± 35.6*	312.5 ± 18.6*	265.5 ± 13.2		233.8 ± 13.0		223.1 ± 9.9*	300.0 ± 16.2	295.0 ± 14.3	221.5 ± 8.9*	248.8 ± 11.9		
Proline	Placebo	207.2 ± 36.5	204.4 ± 38.4	205.3 ± 42.9	194.6 ± 39.8*				173.7 ± 31.5*					169.6 ± 32.4*	0.0166	0.4539
	PULSE	145.9 ± 12.0	175.7 ± 12.9*	160.3 ± 13.3	168.3 ± 11.7*		167.4 ± 13.6*		173.9 ± 11.0*			177.5 ± 11.4*		187.8 ± 10.6*	0.0548	0.0769
	BOLUS	147.9 ± 17.9	258.4 ± 31.6*	285.7 ± 33.6*	228.6 ± 21.2*	183.8 ± 18.4*		161.2 ± 15.6		154.6 ± 13.3	242.1 ± 28.6*	228.2 ± 18.3*	171.3 ± 11.8*	171.2 ± 11.5*		
Arginine	Placebo	87.2 ± 4.4	85.3 ± 3.7	84.6 ± 4.5	82.1 ± 3.9				80.6 ± 3.7*					78.6 ± 4.5*	0.0287	0.3230
	PULSE	78.2 ± 3.4	92.4 ± 4.3*	91.1 ± 3.8*	93.9 ± 3.7*		88.5 ± 4.0*		88.7 ± 2.6*			86.3 ± 2.6*		86.6 ± 2.3*	0.0548	0.0769
	BOLUS	82.6 ± 4.3	121.0 ± 8.4*	129.4 ± 7.7*	101.9 ± 4.9*	86.3 ± 3.5		75.5 ± 3.8*		77.3 ± 3.2	106.9 ± 5.8*	97.8 ± 3.9*	78.1 ± 2.7	79.5 ± 3.2		
Tyrosine	Placebo	69.2 ± 3.8	64.5 ± 3.0	60.9 ± 3.6*	57.0 ± 3.2*				53.2 ± 3.2*					54.9 ± 4.9*	0.0374	0.2586
	PULSE	59.5 ± 1.9	67.2 ± 2.9*	62.3 ± 1.9	60.3 ± 1.7		55.5 ± 1.8*		54.1 ± 1.9*			51.6 ± 2.0*		53.2 ± 2.1*	0.2707	<0.0001
	BOLUS	56.4 ± 1.4	87.2 ± 4.8*	96.8 ± 4.0*	77.3 ± 3.2*	61.5 ± 3.0		49.8 ± 1.8*		49.8 ± 1.7*	77.3 ± 4.1*	75.7 ± 2.5*	56.4 ± 2.0	55.2 ± 1.7		
Valine	Placebo	230 ± 11	225 ± 10	216 ± 11*	209 ± 10*				210 ± 9*					196 ± 9*	0.0414	0.2338
	PULSE	229 ± 9	282 ± 13*	261 ± 9*	276 ± 10*		292 ± 11*		321 ± 15*			328 ± 15*		359 ± 15*	0.4382	<0.0001
	BOLUS	212 ± 9	389 ± 28*	447 ± 23*	382 ± 17*	304 ± 15*		264 ± 12*		261 ± 12*	404 ± 26*	412 ± 15*	321 ± 17*	310 ± 11*		
Methionine	Placebo	22.7 ± 1.0	21.5 ± 0.9	20.6 ± 1.0*	20.3 ± 0.9*				20.9 ± 1.0*					21.6 ± 0.9	0.0199	0.4125
	PULSE	22.0 ± 1.4	27.6 ± 1.3*	23.7 ± 0.8	24.6 ± 0.9		24.0 ± 0.9		25.0 ± 1.2*			23.7 ± 1.0		25.0 ± 1.1*	0.0066	0.5439
	BOLUS	20.0 ± 0.6	46.0 ± 4.4*	48.7 ± 3.5*	32.8 ± 2.1*	22.6 ± 1.5		18.1 ± 1.1		19.9 ± 0.9	38.8 ± 3.1*	33.6 ± 1.7*	21.8 ± 1.1	21.4 ± 0.9		
Isoleucine	Placebo	57.6 ± 2.9	54.9 ± 2.5	50.8 ± 2.7*	49.2 ± 2.3*				53.6 ± 3.0					54.8 ± 2.7	0.0629	0.1400
	PULSE	57.4 ± 2.5	103.6 ± 8.3*	86.0 ± 5.4*	102.2 ± 3.4*		116.1 ± 5.3*		138.8 ± 8.0*			140.8 ± 9.0*		156.7 ± 5.4*	0.6112	<0.0001
	BOLUS	53.5 ± 3.3	201.8 ± 19.5*	247.6 ± 19.7*	171.4 ± 13.5*	115.2 ± 9.7*		84.6 ± 6.1*		81.1 ± 4.8*	203.8 ± 15.2*	201.6 ± 11.9*	123.6 ± 10.9*	108.0 ± 6.3*		
Leucine	Placebo	110.6 ± 5.3	106.9 ± 4.9	99.0 ± 5.5*	96.0 ± 4.7*				107.2 ± 5.4					104.1 ± 5.3	0.0444	0.2173
	PULSE	111.4 ± 4.5	174.2 ± 10.8*	147.4 ± 5.7*	168.2 ± 6.7*		185.3 ± 6.7*		218.4 ± 11.6*			220.4 ± 11.3*		249.1 ± 11.2*	0.6039	<0.0001
	BOLUS	106.4 ± 5.2	301.0 ± 25.6*	368.5 ± 25.6*	264.4 ± 17.8*	180.5 ± 12.4*		147.4 ± 8.4*		146.0 ± 7.6*	311.7 ± 22.8*	303.2 ± 11.8*	193.8 ± 16.2*	178.8 ± 11.2*		

		0 h	0.5 h	1 hour	2 h	3 h	4 h	5 h	6 h	7 h	7.5 h	8 h	9 h	10 h	*R* ^2^	*P‐*value
Tryptophan	Placebo	46.6 ± 2.1	47.4 ± 2.4	45.5 ± 1.6	44.0 ± 2.0				46.9 ± 2.3					47.2 ± 3.1	0.0255	0.3522
	PULSE	43.9 ± 2.5	57.6 ± 3.1*	53.6 ± 2.7*	56.1 ± 2.7*		57.5 ± 2.6*		56.3 ± 2.9*			59.5 ± 2.7*		60.5 ± 3.1*	0.0634	0.0566
	BOLUS	41.8 ± 2.1	76.1 ± 4.8*	93.5 ± 4.4*	77.2 ± 3.6*	55.5 ± 4.2*		41.6 ± 2.5		44.9 ± 1.9	76.7 ± 5.4*	87.8 ± 2.4*	64.9 ± 3.2*	54.5 ± 2.3*		
Lysine	Placebo	151.4 ± 6.4	146.8 ± 6.7	145.5 ± 7.0	143.3 ± 6.1*				142.5 ± 5.8*					136.4 ± 6.1*	0.0285	0.3247
	PULSE	137.3 ± 6.2	182.3 ± 9.8*	166.0 ± 9.6*	173.6 ± 6.8*		175.5 ± 8.8*		182.8 ± 8.1*			171.7 ± 6.5*		180.3 ± 6.6*	0.0207	0.2814
	BOLUS	132.8 ± 5.0	282.9 ± 24.3*	310.9 ± 17.7*	223.2 ± 11.8*	160.3 ± 8.6*		132.4 ± 5.9		131.3 ± 5.6	250.1 ± 16.0*	228.2 ± 8.1*	147.7 ± 7.2	145.5 ± 4.9		
Phenylalanine	Placebo	91.2 ± 3.6	82.2 ± 3.1*	76.7 ± 2.7*	70.6 ± 1.9*				66.8 ± 2.3*					66.7 ± 3.5*	0.1540	0.0179
	PULSE	84.6 ± 4.1	85.8 ± 3.9	77.5 ± 3.4*	71.5 ± 2.5*		63.6 ± 2.7*		63.0 ± 2.6*			59.2 ± 2.5*		61.7 ± 2.3*	0.3026	<0.0001
	BOLUS	83.1 ± 4.3	105.2 ± 6.0*	103.2 ± 4.8*	76.1 ± 3.3	61.3 ± 2.3*		56.3 ± 2.4*		59.6 ± 2.0*	80.8 ± 4.0	73.9 ± 2.5*	58.8 ± 1.7*	59.9 ± 1.5*		
BCAA	Placebo	398 ± 17	386 ± 16	366 ± 18*	354 ± 16*				371 ± 17*					355 ± 15*	0.0002	0.9332
	PULSE	402 ± 14	566 ± 32*	500 ± 18*	553 ± 21*		602 ± 23*		689 ± 35*			698 ± 35*		775 ± 32*	0.5498	<0.0001
	BOLUS	375 ± 17	907 ± 78*	1080 ± 76*	828 ± 49*	607 ± 36*		502 ± 26*		493 ± 24*	939 ± 78*	929 ± 41*	646 ± 44*	604 ± 29*		
EAA^a^	Placebo	923 ± 31	899 ± 28	865 ± 32*	839 ± 27*				849 ± 30*					818 ± 31*	0.1540	0.0179
	PULSE	877 ± 15	1148 ± 49*	1047 ± 29*	1125 ± 26*		1168 ± 27*		1276 ± 40*			1263 ± 44*		1369 ± 41*	0.3026	<0.0001
	BOLUS	843 ± 28	1741 ± 138*	1996 ± 126*	1527 ± 74*	1159 ± 50*		977 ± 35*		964 ± 30	1701 ± 121*	1636 ± 63*	1153 ± 55*	1107 ± 27*		
Total AA^a^	Placebo	2559 ± 69	2502 ± 68	2467 ± 82	2394 ± 76*				2384 ± 70*					2314 ± 88*	0.0440	0.2193
	PULSE	2343 ± 52	2756 ± 87*	2616 ± 73*	2691 ± 57*		2713 ± 70*		2827 ± 81*			2783 ± 81*		2927 ± 77*	0.1585	0.0020
	BOLUS	2346 ± 81	3755 ± 263*	4127 ± 247*	3239 ± 125*	2640 ± 101*		2400 ± 84		2385 ± 72	3525 ± 218*	3399 ± 123*	2551 ± 78	2584 ± 42		

a EAA and total AA were calculated without phenylalanine.

**Figure 3:**
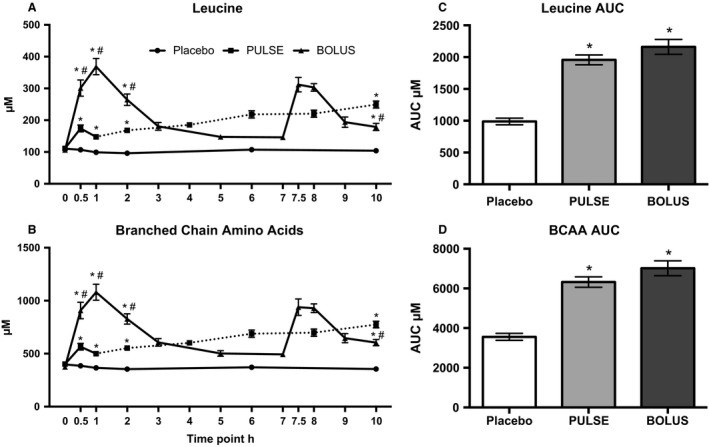
Plasma concentrations of (A) leucine and (B) BCAA measured in postabsorptive state at 0 h and continuously for 10 h in the postprandial phase at the denoted time points. The dotted line represents the phase where peaks in leucine and BCAA levels, as seen at 0.5 h, are expected to occur. The AUC for (C) leucine and (D) BCAA concentrations were calculated from the available time points. *Different from the placebo group. ^#^Different from the PULSE group. Data are shown as the mean ± SEM

Plasma leucine and BCAA concentrations were higher in the PULSE and BOLUS groups than in the placebo group at 0.5, 1, 2, and 10 h (for all the time points, *P* < 0.001 for both the PULSE and BOLUS groups) (Fig. 3A and B). Leucine and BCAA levels measured at 0.5, 1, and 2 h were higher (all *P* < 0.001) in the BOLUS group than in the PULSE group, but when measured at 10 h, the PULSE group had higher concentrations of leucine (*P* < 0.001) and BCAA (*P* = 0.002) than the BOLUS group. Thus, as seen in Figure 3A and B, the group with the highest circulating leucine and BCAA levels shifted during the 10‐h postprandial period. Although the AUCs for the PULSE group were underestimated since blood could not be drawn every half hour, a higher AUC for the leucine concentration was observed in the PULSE (*P* < 0.001) and BOLUS (*P* < 0.001) groups compared with that of the placebo group, and higher AUCs for the BCAA concentration were observed in the PULSE (*P* < 0.001) and BOLUS (*P *< 0.001) groups compared with that of the placebo group (Fig. 3C and D). However, the AUC for leucine (*P* = 0.104) and BCAA (*P *= 0.091) did not significantly differ between the PULSE and BOLUS groups.

### AAT membrane protein expression

**Figure 4:**
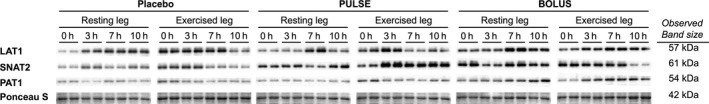
Representative blots from the Western blot analysis of membrane‐associated LAT1, SNAT2, and PAT1 protein expression, and Ponceau‐S staining of the evaluated protein band. Blots are shown for one subject loaded in replicates from each individual group (placebo, PULSE *,* and BOLUS) for the resting and exercised leg at 0, 3, 7, and 10 h. The observed size of the protein bands is indicated for each individual protein target.

**Figure 5:**
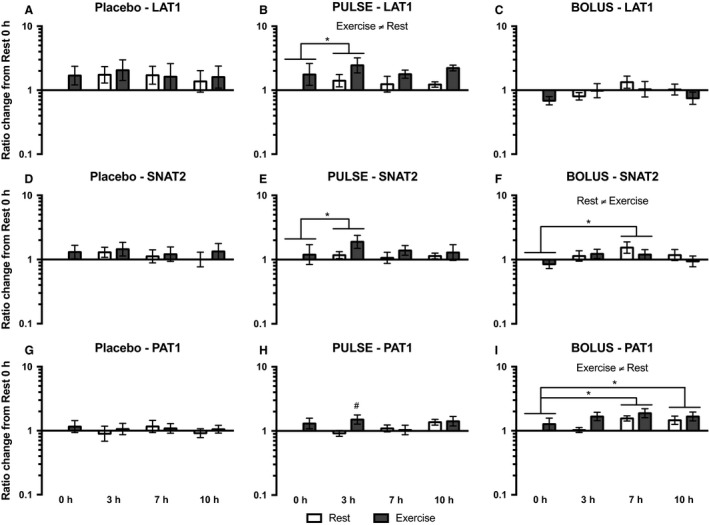
LAT1 (A, B, and C), SNAT2 (D, E, and F)*,* and PAT1 (G, H, and I) protein expression in the placebo, PULSE *,* and BOLUS groups, respectively, for the resting leg (white bars) and exercised leg (dark gray bars) at 0, 3, 7, and 10 h postexercise. Data are expressed as changes from 0 h in the resting leg. Data are shown as the GeoMean ± back‐transformed SEM. *Differences between time points. ^#^Different from the resting leg.

Representative blots from Western blot analyses of the select AATs are shown in Figure 4. In the PULSE group, LAT1 expression was higher in the exercised leg compared with the resting leg during the postexercise period (*P* = 0.011), but no time point‐specific differences were observed (Fig. 5B). At 3 h, the expression was higher compared with that at 0 h (*P* = 0.007), but with no leg‐specific differences observed. In the placebo and BOLUS groups, LAT1 expression did not display significant differences (Fig. 5A and C).

Sodium‐coupled neutral amino acid transporter 2 (SNAT2) expression increased at 3 h compared with that at 0 h in the PULSE group (*P* = 0.018) (Fig. 5E), but with no significant leg differences observed. In the BOLUS group, higher SNAT2 expression was seen in the resting leg than in the exercised leg throughout the postexercise period (*P* = 0.013) (Fig. 5F), and SNAT2 expression was greater at 7 h than at 0 h (*P* = 0.028), but with no leg‐specific differences. Significant differences in SNAT2 expression were not seen in the placebo group (Fig. 5D).

In the PULSE group, PAT1 expression was greater in the exercised leg than in the resting leg at 3 h (*P* = 0.012) (Fig. 5H). For the BOLUS group, PAT1 expression was greater in the exercised than in the resting leg (*P* = 0.049) throughout the whole postexercise period (Fig. 5I), and it was greater at 7 h (*P* = 0.009) and 10 h (*P* = 0.012) than at 0 h, but with no leg‐specific differences. No differences in PAT1 expression were seen in the placebo group (Fig. 5G).

### AAT mRNA expression

**Figure 6:**
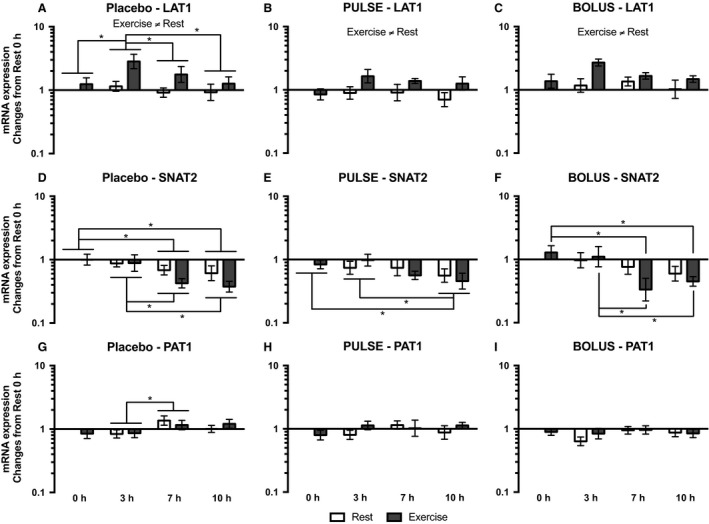
LAT1 (A, B, and C), SNAT2 (D, E, and F), and PAT1 (G, H, and I) mRNA expression for the placebo, PULSE and BOLUS groups, respectively, for the resting leg (white bars) and exercised leg (dark gray bars) at 0, 3, 7, and 10 h postexercise. Data are normalized to RPLP0 mRNA expression and expressed as changes from 0 h in the resting leg. Data are shown as the GeoMean ± back‐transformed SEM. *Differences between time points.

Light‐load resistance exercise increased LAT1 mRNA expression, which was observed as an overall difference between the resting and exercised legs, in the placebo (*P* = 0.020), PULSE (*P* = 0.002) and BOLUS (*P* = 0.004) groups (Fig. 6A, B and C). At 3 h in the placebo group, LAT1 mRNA expression was higher compared with that at 0 (*P* = 0.004), 7 (*P* = 0.026) and 10 h (*P* = 0.001), but it was not significantly related to the exercised leg. In the PULSE and BOLUS groups, only a tendency toward an interaction effect from ANOVA testing was seen (*P* = 0.062 and *P* = 0.083, respectively); thus, no post hoc test could be performed to reveal time point‐specific differences.

Sodium‐coupled neutral amino acid transporter 2 (SNAT2) mRNA expression in the placebo group decreased at 7 (*P* = 0.006) and 10 h (*P* < 0.001) compared with that at 0 h, and at 7 (*P* = 0.024) and 10 h (*P* = 0.003) compared with that at 3 h; no leg‐specific differences were observed (Fig. 6D). In the PULSE group, SNAT2 mRNA expression decreased at 10 h compared with that at 0 (*P* = 0.037) and 3 h (*P* = 0.045), and no leg‐specific differences were observed (Fig. 6E). In the BOLUS group, SNAT2 mRNA expression decreased in the exercised leg at 7 (*P* = 0.001) and 10 h (*P* = 0.018) compared with that at 0 h, and at 7 (*P *= 0.002) and 10 h (*P* = 0.018) compared with that at 3 h (Fig. 6F).

In the placebo group, PAT1 mRNA expression increased from 3 to 7 h (*P* = 0.024), and no leg‐specific differences were observed (Fig. 6G). No significant changes in PAT1 mRNA expression were observed in either the PULSE or BOLUS groups (Fig. 6H and I).

## Discussion

The main purpose of the present study was to investigate the impact of LL‐RE, with or without protein intake, on the expression of membrane‐associated AATs. We demonstrated that LL‐RE, combined with whey protein intake, increases the membrane‐associated protein expression of select AATs. To our knowledge, we are the first to fractionate human muscle homogenates to study exercise and nutrient effects on AAT expression. Whey protein intake primarily resulted in a robust change in plasma BCAA concentrations reflecting the intake pattern, indicating that these amino acids, combined with exercise, could be driving the regulation of AAT protein expression. Moreover, LL‐RE increased LAT1 mRNA expression irrespective of the feeding regimen, whereas SNAT2 mRNA expression decreased over time in all three groups, although only in the exercised leg of the BOLUS group. Because acute changes in the expression of membrane‐associated AATs are very much related to AAT function (Hyde et al. 2001; Hundal and Taylor 2009), we fractionated homogenized skeletal muscle tissue in this study. This allowed us to only observe changes in the expression of membrane‐associated AATs, which provides insight into changes in the potential for amino acid transport and sensing, whereas previous studies have looked at changes in AAT protein expression in whole‐muscle tissue homogenates (Drummond et al. 2010, 2011; Dickinson et al. 2013). However, we should acknowledge that these previous studies have reported the regulation of AATs in young and old subjects in response to RE with (Dickinson et al. 2013) or without (Drummond et al. 2011) a postexercise EAA drink. Dickinson et al. (2013) only saw up‐regulated LAT1 protein expression in old subjects, whereas only young subjects displayed an increase in SNAT2 protein expression. Drummond et al. (2011) only saw up‐regulated LAT1 protein expression in young subjects. Thus, both studies indicate that aging affects the AAT response. Despite differences in study design, the increased expression of membrane‐associated LAT1, in response to exercise and frequent protein intake in elderly subjects in the present study (Fig. 5B), is in line with previous findings that showed an increase in LAT1 expression in a whole‐muscle homogenate in response to heavy‐load RE and EAA intake (Dickinson et al. 2013). An increase in membrane‐bound LAT1 could facilitate a greater capacity for influx of leucine, which is important for mTORC1 activation. Moreover, Dickinson et al. (2013) did not observe any change in whole‐muscle cell SNAT2 protein expression in elderly subjects, which is in line with the membrane‐associated SNAT2 protein expression in the present study, where only a time course effect was seen in the PULSE (Fig. 5E) and BOLUS groups (Fig. 5F). Moreover, we showed that PAT1 membrane‐associated protein expression increased in response to LL‐RE with PULSE or BOLUS protein intake. Such an increase in lysosomal membrane‐bound PAT1 would increase the capacity for mTORC1 docking and subsequent activation at the lysosomal membrane.

The membrane‐associated AAT expression response to LL‐RE and protein ingestion seems to be affected by the protein ingestion regimen, frequent or bolus. Direct comparisons of groups, legs, and time with a three‐way ANOVA have not been made, as the sample size was too small to perform a reliable test. Thus, group comparisons were evaluated qualitatively, discussing the changes seen within each group individually. The clearest distinction between groups was seen in LAT1 protein expression, where an overall increase in expression was seen in the exercised leg compared with that of the resting leg in the PULSE group, whereas neither exercise‐induced nor time‐dependent changes were seen in the BOLUS group. The distinct LAT1 protein expression between the PULSE and BOLUS groups could be a matter of supply and demand. Most likely, the intracellular AA pool was depleted more rapidly in the PULSE group than in the BOLUS group in the first 3 h of the postprandial phase because of lower plasma AA concentrations (Table 3), caused by a different total amount of protein consumed until this time point; PULSE 12 g and BOLUS 28 g of whey protein. Thus, the early (3 h) increased LAT1 and also SNAT2 protein expression in the PULSE group could increase the potential for repletion of intracellular BCAAs, thus, matching the demand. Unfortunately, due to a lack of muscle tissue for intracellular AA measurement, we do not have data on intracellular AA concentrations. For the BOLUS group, a substantial increase in the plasma AA concentration was seen after the first protein bolus (Table 3). The very quick increase in circulating amino acids and the concomitant increase in insulin (Agergaard et al. 2017) after ingesting the whey proteins were presumably sensed immediately by the peripheral muscle cells, which could facilitate a dose‐dependent increase in the amino acid flux into the muscle cells (Biolo et al. 1995, 1997). Thus, the supply of AA in the BOLUS group could be sufficient without the need for increasing membrane‐associated LAT1 protein expression to match the intracellular need for AA in the initial 3‐h period. Instead, SNAT2 and PAT1 protein expression increased at 7 h in the BOLUS group. This increased AAT expression could be caused by low plasma AA concentrations in the BOLUS group at this point (Fig. 3 and Table 3), to match a possible lower supply of AAs.

One important limitation of the membrane fractionation technique of the muscle homogenates is that it does not distinguish between different membranes (e.g., plasma, nuclear, or lysosomal membranes). Thus, we cannot definitely state the site at which the membrane‐associated AAT expression changed. We only showed that the capacity for AA transport of the muscle cells changed, and along with that suggest that the changes in the expression of membrane‐associated LAT1 and SNAT2 would occur at the plasma membrane, while that of PAT1 would occur at the lysosomal membrane, where these AATs have been shown to have an important effect on mTORC1 activation (Poncet and Taylor 2013). Future laboratory approaches, for example, microscope techniques, could potentially illustrate specific AAT translocations and site‐specific AAT expression in response to interventions such as exercise and protein intake.

Reverse transcription‐PCR was performed to analyze AAT mRNA expression. This would elucidate the potential for de novo AAT synthesis, while the expression of membrane‐associated proteins showed the immediate capacity for amino acid flux. The expression of LAT1 mRNA increased in response to LL‐RE, irrespective of the feeding regimen. This indicates the importance of LAT1 in the anabolic response to resistance exercise, although over the course of 10 h we only saw this translated into increased membrane‐associated protein expression in the PULSE group. SNAT2 mRNA expression decreased over time in all three groups, and in the BOLUS group, this effect was significant only for the exercised leg. We do not believe that this effect can be caused by the increased AA availability, as decreased SNAT2 mRNA expression was also observed in the placebo group; nor can it solely be ascribed to a circadian effect, as in the BOLUS group the decrease was merely seen in the exercised leg. Notably, we observed similar mTORC1 signaling effects, especially for eukaryotic elongation factor 2 (eEF2) (Agergaard et al. 2017), but, a connection require further research. Decreased SNAT2 transporter activity would decrease intracellular glutamine transport, which will inhibit the capacity for intracellular BCAA uptake, thus tapering AA‐driven mTORC1 activation (Nicklin et al. 2009). The increased LAT1 mRNA expression is in line with previous findings on the responses to heavy‐load RE in the elderly (Drummond et al. 2011; Dickinson et al. 2013). In contrast, the present study is the first to show a decrease in SNAT2 mRNA expression from the baseline to late into the postexercise period. The studies by Drummond et al. (2011) and Dickinson et al. (2013) showed an increase in SNAT2 mRNA expression 3 h into the postheavy‐load exercise period, which returned to basal levels at 6 h. The increased SNAT2 mRNA expression at 3 h could be an effect of the heavy‐load RE in those studies compared with the lack of an effect at 3 h in response to LL‐RE in the present study. The need to maintain greater hypertrophy signaling in response to heavy‐load RE could be the significant difference compared with the present study.

For the two whey protein groups (PULSE and BOLUS), the exact same amount of protein (700 mg whey protein × kg FFM^−1^, or approximately 40 g of whey protein in total) was ingested throughout the 10‐h postexercise period. Although underestimated for the PULSE group, we also observed the same AUC for the leucine and BCAA concentrations for the PULSE and BOLUS groups. The PULSE strategy was chosen to maintain an elevated availability of AAs for a prolonged period of time and, thereby, to copy the previous study design of Bechshoeft et al. (2013), whereas the strategy with two boluses was chosen as a more applicable approach toward protein intake, while still equalizing the total amount of protein ingested throughout the study. Because of the different protein ingestion patterns (PULSE vs. BOLUS) (Fig. 1), differences in plasma AA concentrations were seen; BOLUS feeding causing two spikes in plasma AA concentrations, whereas PULSE feeding resulting in a small but prolonged increase in plasma AA concentrations (Table 3). Thus, the different responses between the PULSE and BOLUS groups were not caused by different whey protein amounts in the total 10‐h postprandial period, but by the different feeding regimens causing different plasma AA concentrations.

The cause of age‐related deterioration of skeletal muscle mass (Janssen et al. 2000) is believed to be multifactorial (Morley et al. 2011), but several studies have documented anabolic effects of resistance exercise and amino acid administration (either in the form of whole protein, BCAAs, or EAAs) in elderly subjects (Kosek et al. 2006; Dideriksen et al. 2011; Symons et al. 2011; Yang et al. 2012), thus proposing a way to diminish the aging process of skeletal muscle. The effects of resistance training and hyperaminoacidemia result in a positive muscle protein net balance, which is mainly caused by an increase in MPS. Elsewhere, we have shown that LL‐RE in elderly subjects results in greater MPS throughout the 10‐h postexercise period, irrespective of placebo or protein (PULSE or BOLUS) intake (Agergaard et al. 2017). Notably, we only saw a small effect of protein intake. However, as shown in the present study, LL‐RE, together with protein intake, seemed to potentiate anabolic mechanisms that make skeletal muscle more susceptible to AAs. This effect was especially evidenced by increased LAT1 protein expression throughout the entire 10‐h postexercise period in the PULSE group, which increased the capacity for leucine uptake. An adequate supply of AAs for protein synthesis is known to be important for the anabolic response (Biolo et al. 1997, 1999). Therefore, we believe that the ability of an exercise regimen to increase the expression of AATs is important, as it makes the skeletal muscle more susceptible to AA availability and, thus, may make the muscle more anabolic potent. Previously, it has been shown that in the absence of previous exercise, elderly subjects responded less to a given amount of EAAs (Cuthbertson et al. 2005). However, resistance exercise prior to protein or EAA intake has been shown to induce the same increase in MPS in young and elderly subjects (Drummond et al. 2008; Pennings et al. 2011). Therefore, resistance exercise, which induces an increase in AAT protein expression, could shift the leucine threshold (Breen and Phillips 2011), thereby causing a greater anabolic response to protein ingestion. However, long‐term studies are needed to elucidate whether resistance training with a light load increases the basal expression of AATs and whether this translates into a greater AA sensing and, thereby, counteracts anabolic resistance to protein ingestion.

## Conclusion

Light‐load RE followed by whey protein intake increased the expression of membrane‐associated LAT1 and PAT1 in elderly subjects. Different feeding regimens, PULSE or BOLUS, which induced different levels and patterns of, especially, plasma BCAA concentrations, affected the expression of AATs, as increased LAT1 protein expression was only seen following LL‐RE and PULSE feeding, whereas SNAT2 and PAT1 protein expression increased in the BOLUS group in a period with a low plasma BCAA concentration. This suggests that plasma BCAA concentrations could affect membrane‐associated AAT expression. LL‐RE induced an increase in LAT1 mRNA expression, suggesting the importance of LAT1 in the anabolic response to resistance exercise. In contrast, SNAT2 mRNA expression decreased over time. The effect of LL‐RE on AAT protein and mRNA expression points toward an anabolic effect of LL‐RE, although future studies must clarify how the present acute response will translate into long‐term changes.

## Conflict of Interest

None of the authors has any conflict of interest.
